# An evaluation of neurocognitive models of theory of mind

**DOI:** 10.3389/fpsyg.2015.01610

**Published:** 2015-10-31

**Authors:** Matthias Schurz, Josef Perner

**Affiliations:** Centre for Cognitive Neuroscience, University of SalzburgSalzburg, Austria

**Keywords:** theory of mind, mentalizing, neurocognitive theories, meta-analysis, task-categories, temporoparietal junction, medial prefrontal cortex

## Abstract

We review nine current neurocognitive theories of how theory of mind (ToM) is implemented in the brain and evaluate them based on the results from a recent meta-analysis by Schurz et al. (2014), where we identified six types of tasks that are the most frequently used in imaging research on ToM. From theories about cognitive processes being associated with certain brain areas, we deduce predictions about which areas should be engaged by the different types of ToM tasks. We then compare these predictions with the observed activations in the meta-analysis, and identify a number of unexplained findings in current theories. These can be used to revise and improve future neurocognitive accounts of ToM.

## Introduction

For nearly two decades, Theory of Mind (ToM) has been studied in hundreds of functional neuroimaging studies and competing theories about certain brain areas supporting specific cognitive sub-processes of mentalizing exist. Understanding the cognitive processes that are underlying typical ToM, and knowing in which situations they come into play, provides an important interpretational basis for findings of atypical ToM in developmental and psychiatric disorders.

Most neurocognitive theories converge on the following definition of ToM: the ability to make inferences about one’s own and other people’s mental states. However, very diverse tasks are used to operationalize ToM, and different theories often rely on findings from different studies, which influences the scope of these accounts. Increasing evidence shows that the neural signature of ToM differs for different tasks and stimuli (e.g., Gobbini et al., 2007; Bahnemann et al., 2010; Schurz et al., 2014). Therefore, it was argued (Schaafsma et al., 2015) that ToM should not be treated as monolithic ability in brain research, but needs to be deconstructed into more basic sub-processes which allow a more specific mapping to brain areas. The key for such a deconstruction is to know – or to have a good hypothesis about – which are the underlying sub-processes to look at.

One promising way to define the sub-processes of ToM would be a cognitive ontology, like the cognitive atlas (Poldrack et al., 2011; visit http://www.cognitiveatlas.org). To date, cognitive ontologies are in the build-up, but a definition of sub-processes involved in ToM is still missing. In the present review, we rely on neurocognitive theories that make different hypotheses about the sub-processes of ToM.

We review nine neurocognitive theories on ToM, and summarize which sub-processes (i.e., forms of cognitive processing) are supposed to be engaged in ToM. We also review how these sub-processes are thought to link to brain areas, and formulate predictions about whether these processes/areas should be engaged by the demands of different ToM tasks. Predictions from theory are then evaluated based on the results from our meta-analysis (Schurz et al., 2014).

For practical reasons, we only address some of the most popular representatives of an immensely large field of published neurocognitive theories and, in addition, we focus our review on two major brain areas for ToM – the temporo-parietal junction (TPJ) and the medial prefrontal cortex (mPFC).

## Meta-Analysis Fractionating ToM

**FIGURE 1:**
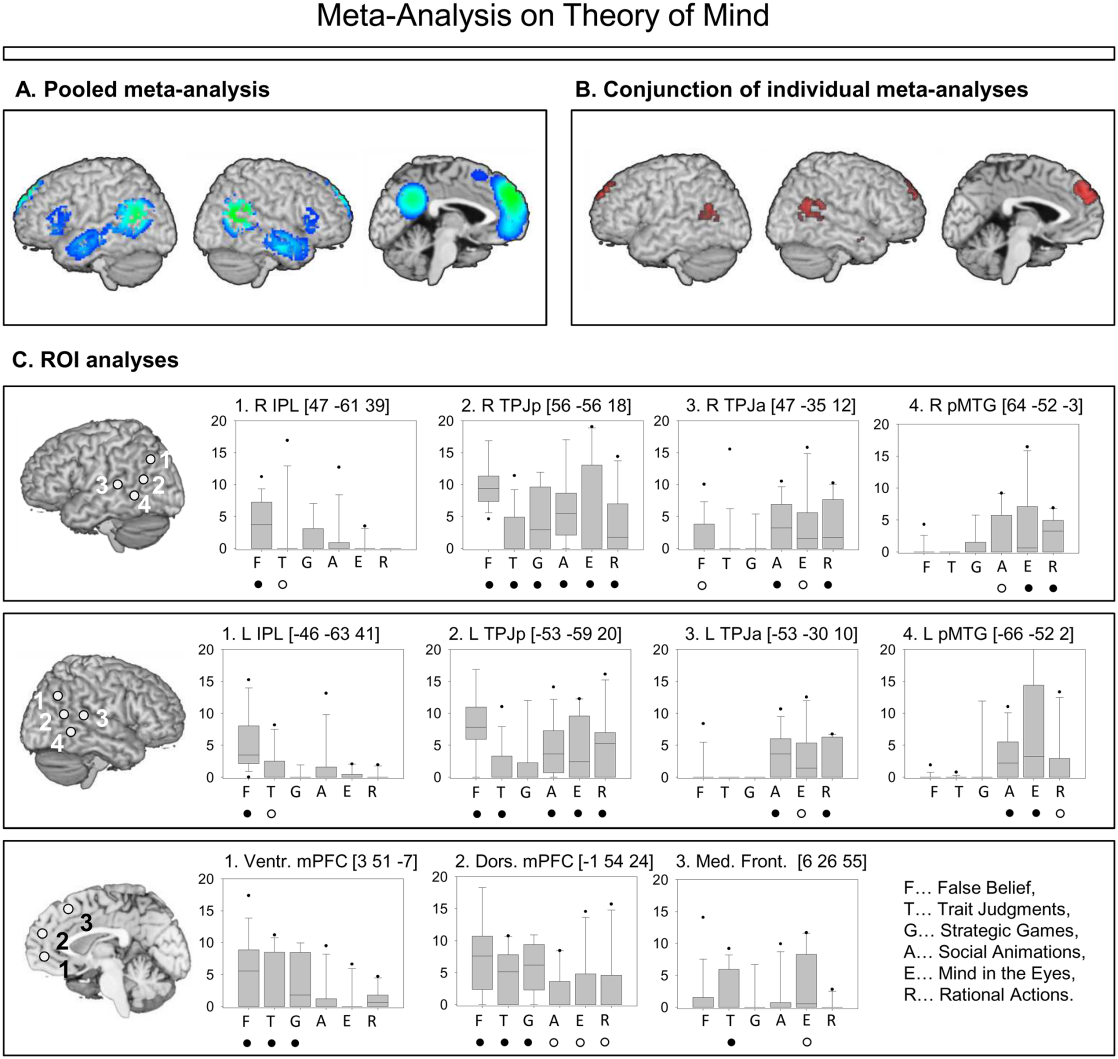
**Summary of the results in Schurz et al. (2014). (A)** Pooled meta-analysis on Theory of Mind (ToM) across all task-groups. Colors represent probability values from statistical permutation testing (*z*-values). **(B)** Conjunction of six meta-analyses, statistically powerful permutation-based overlap analysis (for details, see Schurz et al., 2014). Maps were thresholded at voxel-wise threshold of *p* < 0.005 uncorrected and a cluster extent threshold 10 voxels. **(C)** Regions of interest in posterior temporo-parietal and medial prefrontal areas. Box-plots (median; 25 and 75th percentiles; 5 and 95th percentiles) show the distributions of effect-sizes for the studies in each group. Effect-sizes were weighted by intra-study variances. Significant convergence of effect-sizes above zero was determined by randomization tests; full circles indicate *p* < 0.005 uncorrected, z > 1. Empty circles indicate *p* < 0.05, z > 1.

Schurz et al. (2014) looked at the most common tasks in the neuroimaging literature on ToM, and identified six large task groups. We give representative examples for these tasks in **Table 1**. When pooling brain activation over task groups, the meta-analysis found the typical mentalizing network described in the literature (**Figure 1A**). However, after performing separate meta-analyses for each task group (**Figure 1B**), convergence activation across tasks was found only in bilateral TPJ posterior (TPJp) and dorsal mPFC. The task specific activation patterns were then captured by ROI analyses, which are shown in **Figure 1C**. The TPJ ROIs were placed into different sub-areas based on results from a connectivity-based parcellation (Mars et al., 2011, 2012, 2013) of that area: More dorsal/posterior ROIs in the Inferior Parietal Lobule (IPL) and posterior TPJ (TPJp), and more anterior/ventral ROIs in the anterior TPJ (TPJa) and the posterior Middle Temporal Gyrus (pMTG). Furthermore, several ROIs were similarly placed in the mPFC according to a connectivity-parcellation (Sallet et al., 2013): a ventral mPFC ROI (in so-called connectivity cluster #4), and a dorsal mPFC ROI (connectivity cluster #3), as well as a posterior frontal cortex ROI (in connectivity cluster #2). Locations of these ROIs are indicated in **Figure 1C**.

**Table 1:** Examples from each task-group in the meta-analysis by Schurz et al. (2014).

Author	Imaging	Experimental task	Control Task
		**False belief vs. photo**	
Saxe and Kanwisher, 2003	fMRI *n* = 21	Read a short vignette involving a person holding a false belief. Answer a question about her belief. e.g., *John told Emily that he had a Porsche. Actually, his car is a Ford. Emily doesn’t know anything about cars so she believed John. When Emily sees John’s car, she thinks it is a …? (Porsche or Ford).*	Read a false-photograph vignette. Answer a question concerning the outdated content in the photo. e.g., *A photograph was taken of an apple hanging on a tree branch. The film took half an hour to develop. In the meantime, a strong wind blew the apple to the ground. The developed photograph shows the apple on the …? (tree or ground).*
		**Trait judgments**	
Mitchell et al., 2002	fMRI *n* = 34	Read an adjective. Indicate whether it can be true for a hypothetical person. e.g., “*nervous” … can it be true for “David?”?*	Read an adjective. Indicate whether it can be true for an object. e.g., “*sundried” … can it be true for “grape”?*
		**Strategic games**	
Kircher et al., 2009	fMRI *n* = 14	Play the prisoner’s dilemma game (iterated version). You play with a human player for game points. Both players choose a cooperative or defective strategy on each trial. If both players choose defective, they gain almost no game points at all. If both choose cooperative, both gain some game points. If players choose differently, the defective player gains more points.	Play the prisoner’s dilemma game (iterated version). You play with a computer.
		**Social animations**	
Castelli et al., 2000	PET *n* = 6	Watch a video animation of two interacting triangles (e.g., *mother and child are playing*). Explain verbally what was happening (after fMRI).	Watch video animation of two randomly moving triangles. Explain verbally what was happening (after fMRI).
		**Mind in the eyes**	
Baron-Cohen et al., 1999	fMRI *n* = 12	View photographs of eyes. Indicate which of two words (e.g., *concerned* vs. *unconcerned*) describes the mental state of that person.	View photographs of eyes. Indicate if the person is male or female.
		**Rational actions**	
Brunet et al., 2000	fMRI *n* = 8	View a cartoon story and predict what will happen based on intentions of a character (no false belief). Choose a logical story ending from several options shown in pictures. e.g., *A prisoner is in his cell. First, he breaks the bars of his prison window. Then he walks to his bed. Participants must indicate what will happen next … the prisoner ties a rope from the sheets on his bed/the prisoner shouts out loud.*	View a cartoon story and predict what will happen based on physical causality. Choose a logical story ending from several options shown in pictures. e.g., *A person is standing in front of a slide. A large ball is coming down this slide, heading toward the person standing there. Participants must indicate what will happen next … the ball is knocking over the person/the ball is resting on the ground and the person is standing next to it.*

## Comparing Predictions From Neurocognitive Accounts to Results of the Meta-Analysis

Results from our meta-analysis – with a focus on ROI results shown in **Figure 1C** – will be related to different neurocognitive theories. On the one hand, we will review theories that assume that areas have a ToM-specific function. On the other hand, we will review theories that assume a domain-general function of these areas, which are supporting ToM among other cognitive abilities. The predictions of these proposals and their fit to the data from our meta-analysis are summarized in **Table 2**, and will be discussed in the following sections in detail.

**Table 2:** Summary of evaluation of neuro-cognitive ToM accounts based on our results.

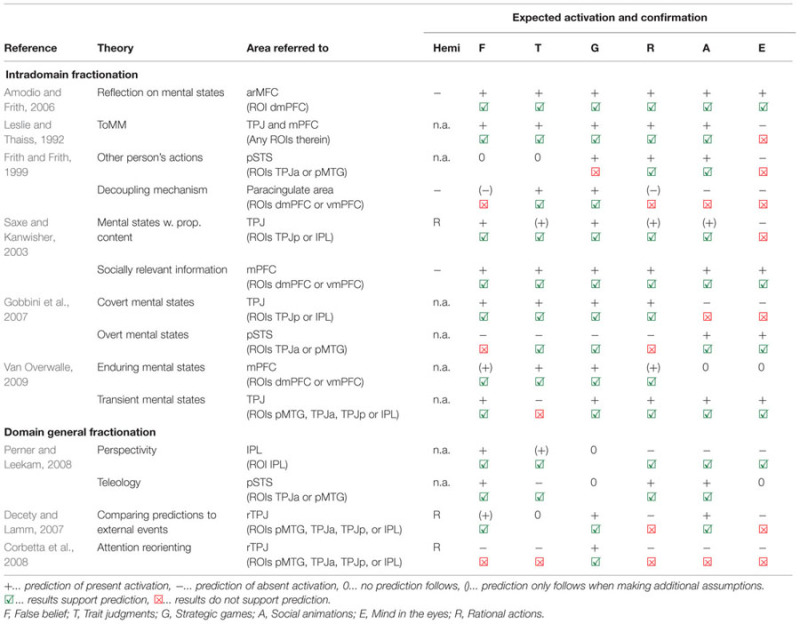

### Domain Specific Theories

#### Theory of Mind Mechanism

*Theory.* Leslie and Thaiss (1992) argued for the existence of ToM mechanism in the brain (ToMM), which is responsible for “kick-starting belief and desire attribution” (Leslie et al., 2004, p. 528).

*Predictions.* Leslie and Thaiss’s (1992) theory makes no prediction about the location of the ToMM, so we assume it could be found both in the TPJ and in the mPFC. We predict the ToMM to be involved in the tasks false belief, trait judgments, strategic games, rational actions and social animations, because they all implicate processing of beliefs and/or desires. We predict ToMM not to be involved in the mind in the eyes task, as it does not provide any information regarding beliefs or desires.

*Evaluation.* Both TPJ and mPFC show activation for all five tasks where we predicted it (see **Table 2** for summary). However, TPJ and mPFC also show activation for the mind in the eyes task, which is inconsistent with the ToMM hypothesis.

#### mPFC for Mental State Reflection

*Theory.* Amodio and Frith (2006) suggested that any kind of reflection about mental states activates the anterior rostral region of the Medial Frontal Cortex (arMFC), which roughly corresponds to the location of our dorsal mPFC ROI.

*Predictions.* All six types of tasks should activate the dmPFC.

*Evaluation.* We indeed find activation for all tasks in the dmPFC ROI.

#### pSTS for Actions and mPFC for Decoupling

##### pSTS

*Theory.* Frith and Frith (1999) proposed a system for representing other person’s actions in the posterior Superior Temporal Sulcus (pSTS).

*Predictions.* Information about other’s actions is presented in the tasks rational actions, strategic games and social animations (for the latter, movements trigger the perception of actions), so we expect pSTS involvement here. No clear prediction follows for false belief and trait judgements, since false beliefs may trigger anticipation of mistaken actions, and traits may involve habitual action tendencies. No actions are presented in the mind in the eyes task, so we expect no activation in the pSTS here.

*Evaluation.* To our knowledge, there is no clear anatomical differentiation between TPJ and pSTS, but it is largely agreed that TPJ is located more dorsal/posterior compared to pSTS. We will therefore use dorsal/posterior ROIs in TPJp and IPL as proxy for TPJ, and ventral/anterior ROIs in TPJa and pMTG as proxy for pSTS. Confirming the predictions, pSTS is activated for rational actions and social animations. Contrary to our predictions, no activation showed in pSTS when it was expected for strategic games, but activation was present for mind in the eyes where it was not predicted.

##### mPFC

*Theory.* Gallagher and Frith (2003) suggested that the paracingulate cortex (roughly corresponding to our ROIs dmPFC and vmPFC) hosts the decoupling mechanism proposed by Leslie (1987), which enables keeping separate representations from their ordinary input–output relations. This is necessary for representing anything that is not straight registration of reality, such as pretense, false beliefs, and, presumably, photographs. Note that decoupling actually is a domain-general computational mechanism, so it could be grouped to the theories presented in the next section.

*Predictions.* Trait judgments and strategic games require hypothetical considerations and thus a decoupling mechanism. So do rational actions and false belief tasks, but here the control conditions used in studies are of particular relevance: for rational action tasks, control conditions require causal reasoning about physical events, and for false belief, they ask about the content shown on an outdated photograph. Thus, both control conditions require hypothetical thinking just as the corresponding experimental conditions of the tasks, so we do not expect to find (relatively increased) brain activation in the mPFC. Finally, for processing social animations and mind in the eyes tasks no possibilities have to be considered, and so no decoupling and mPFC activation is expected.

*Evaluation.* In keeping with our predictions, trait judgments and strategic games elicited activation the mPFC. Contrary to our predictions, we also observed activation for the other four task groups in the mPFC (at least at an uncorrected threshold in the dmPFC ROI), where we expected to see none.

#### TPJ for Beliefs and mPFC for Socially Relevant Information

##### TPJ

*Theory.* Saxe and Kanwisher (2003; see also Kanwisher, 2010) suggested that right TPJ (which the authors locate dorsal/posterior to pSTS) is representing mental states with propositional content like thoughts and beliefs, but not other mental states without propositional content (like feelings or bodily sensations).

*Predictions.* False belief and strategic games tasks require reasoning about what another person thinks is going to happen, so we expect them to activate the right TPJ. The case is less clear for most other task groups. Saxe (2006) suggested that belief-desire reasoning is also needed for thinking about true beliefs in connection with intentional actions. If we accept this additional assumption, we can predict that social animations and rational actions also activate the right TPJ. Furthermore, traits can be viewed as habitual patterns of behavior, thought, and emotion. From this perspective, we can also expect trait judgments to activate the TPJ. Finally, we predict that the mind in the eyes does not activate the TPJ, as it does not require thinking of beliefs but rather judging about feelings (without propositional content, e.g., judging that the person seems concerned, but not making assumptions about what the person is concerned about).

*Evaluation.* We found activation in the right TPJ, in particular in the ROI TPJp, for all five tasks were we expected it. However, our prediction of absence of activation for the mind in the eyes task was not fulfilled, as this task also elicits activation in the right TPJ.

##### mPFC

*Theory.* Saxe and Powell (2006, see also Saxe and Wexler, 2005) suggested that the mPFC has a less specific role in ToM, and is engaged whenever we are processing socially or emotionally relevant information about others.

*Predictions.* As all ToM tasks obviously present socially and emotionally relevant information about others, we predict that mPFC shall be engaged in all tasks.

*Evaluation.* Our meta-analysis fully supports this prediction, as all tasks activate in mPFC, in particular in the dmPFC.

#### TPJ for Covert and pSTS for Overt Mental States

##### TPJ

*Theory.* Gobbini et al. (2007) found that ToM tasks involving false beliefs activate the TPJ more dorsally than social animations and point-light-movement tasks. They hypothesized that this reflects the difference between covert mental states that need to be inferred from what one observes (e.g., beliefs) and more overt mental states, like intention-in-action (Searle, 1980), where one can perceive the mental states in the observed movements.

*Predictions.* From the theory that covert mental states activate in TPJ/IPL, we predict that false belief, trait judgments, strategic games and rational action tasks should activate the area. All four tasks present covert mental states in the sense that what needs to be represented is not immediately observable from an action. Social animations and mind in the eyes tasks, on the other hand, ask for inference about mental states which manifest in a movement or facial expression, so we predict no activation here.

*Evaluation.* The predicted activation for the abovementioned four tasks was found. However, predictions of absent activation for social animations and mind in the eyes were not supported. We found also here activation in TPJ, namely right TPJp (but not IPL).

##### pSTS

*Theory.* Gobbini et al. (2007) hypothesized that overt mental states activate more ventral areas in pSTS. We take ROIs in TPJa and pMTG as a proxy for that location.

*Predictions.* Predictions are the opposite from those made above, that is, pSTS should not be engaged by false belief, strategic games, trait judgments and rational action tasks. However, the area should be engaged by social animations and mind in the eyes, as these present overt mental states.

*Evaluation.* The predicted occurrence of activation for social animations and mind in the eyes tasks was supported by our results. Also in keeping with our predictions, no activation was found for trait judgments and strategic games. However, different from what we expected, also false belief and rational action tasks showed activation in pSTS (in particular in right TPJa).

#### TPJ for Transient and mPFC for Enduring Mental States

##### TPJ

*Theory.* According to Van Overwalle’s (2009) model, the bilateral TPJ (including pSTS, TPJ and IPL areas) is engaged in making inferences about transient mental states such as immediate goals and desires.

*Predictions.* In the tasks false belief, strategic games, rational actions, social animations and mind in the eyes the goal is to infer a transient mental state. For trait judgments, we predict absence of activation, since no immediate goals or desires are involved.

*Evaluation.* We found activation in the TPJ (broadly defined by the model as pSTS, TPJ and IPL) for all five task groups where this was predicted. However, activation was also found for trait judgment tasks where we predicted no activation.

##### mPFC

*Theory.* Van Overwalle (2009) hypothesized that the mPFC is engaged in making inferences about permanent social and psychological properties of others, such as personality traits.

*Predictions.* Information about enduring mental states is cleary processed in trait judgment tasks, and so mPFC activation is expected. Following Van Overwalle’s (2009) reasoning, we also predict activation for strategic games, because each player must develop an impression of the trustworthiness, cooperativeness, or competitiveness of the other. Another prediction following Van Overwalle (2009) is that tasks presenting a rich social context in their stimulus material could lead to spontaneous trait inference, and thus engage the mPFC. Based on this assumption, we furthermore expect activation in mPFC for false belief and rational actions. With respect to social animations and mind in the eyes tasks, Van Overwalle’s (2009) theory makes no clear predictions.

*Evaluation.* In agreement with our prediction, we found activation in mPFC for false belief, trait judgments, strategic games, and rational actions. We also found activation for the two task groups where we made no predictions.

### Domain General Theories

#### pSTS for Teleology and IPL for Perspective

##### pSTS

*Theory.* Perner and Leekam (2008) and Perner and Roessler (2010, 2012) proposed two cognitive mechanisms for ToM: teleology and perspective taking (appreciation of perspective differences). Teleology (coming from the greek word *telos* which stands for *purpose* or *goal*) is linked to the pSTS and means applying means-ends reasoning in order to predict others actions, i.e., an agent will do what is needed in given circumstances. No belief-desire reasoning requiring an appreciation of different perspectives is involved in this form of behavior explanation.

*Predictions.* The social animation and rational action tasks can be interpreted within teleology. Also for false belief tasks, applying the principle of rationality is required (however, it must be put into perspective, see next section). No clear prediction can be made for mind in the eyes and strategic games. In strategic games, this is due to the nature of the control condition: players need to consider what the other player is going to do, which may involve means-ends reasoning. However, this may also take place during the control condition playing against a computer algorithm, where one also needs to figure out the computer’s strategy and goals (see also Schurz and Tholen, 2015 for discussion). For trait judgments, no immediate goals, circumstances or actions are relevant, and so no teleology and activation in pSTS is predicted.

*Evaluation.* Results fully support our predictions: activation was present for social animations, rational actions and false belief tasks (for the latter only in right TPJa), and absent for trait judgment tasks.

##### IPL

*Theory.* The second process to understand others is perspective taking linked to IPL (dorsal TPJ). This allows belief-desire reasoning by considering agents’ representations and subjective perspectives of what is needed. The mental subjectivity of other people’s reasoning can then be captured by teleology within the other’s perspective (‘teleology-in-perspective’), i.e., in the case of a false belief the other person will do what she would need to do if the world were as she thinks it is. Processing perspectivity is a process that cuts across the domain of ToM to other areas of knowledge such as understanding false signs (Aichhorn et al., 2009), identity (Arora et al., 2015), or level 2 visual perspective taking (Schurz et al., 2013; see also Schurz et al., 2015).

*Predictions.* No perspective taking is needed for social animations, mind in the eyes, or rational actions, and so no activation of IPL is expected. False belief tasks require means-ends reasoning (teleology) from another person’s perspective, so we predict activation in IPL here. For similar reasons as mentioned before in the section on pSTS, no clear prediction can be made for strategic games. Trait judgments, on the other hand, may require awareness of perspective. Traits are habitual patterns of behavior, thought, and emotion. They are characteristic for a person when the person’s habits deviate from the norm. For instance, a person is called “anxious” or “nervous” (Mitchell et al., 2002) if she tends to be concerned about situations where one normally has no reason to be anxious. Therefore, many trait judgments are judgments about whether a person habitually takes a different perspective on certain things in life, and so we tentatively predict trait judgments to activate IPL.

*Evaluation.* Our predictions were fully supported. We only found activation for false belief and trait judgment tasks in IPL, but not for strategic games, social animations, rational actions, and mind in the eyes tasks.

#### TPJ for Predictions about External Events

*Theory.* According to Decety and Lamm (2007, p. 583), ToM tasks engage a domain-general “lower-level computational mechanism involved in generating, testing, and correcting internal predictions about external sensory events”, which is mediated by the right TPJ.

*Predictions.* For simplicity, we assume that only generating a prediction about an external event already triggers activation in the TPJ. We expect activation for strategic games and social animations which obviously require generating predictions about an upcoming event. For false belief tasks we can also expect activation, if we additionally assume that participants automatically think of the behavioral consequences of the protagonist’s false belief. No activation in TPJ can be expected for mind in the eyes, because they do not refer to external events, and for rational actions because here studies asked participants to predict an external event both in the experimental (e.g., predict what the person will do next) and in the control condition (e.g., predict what will happen due to physical causality). For trait judgments, no clear expectation follows.

*Evaluation.* Consistent with our predictions we found activation for false belief, strategic games, and social animations. Inconsistent with our predictions, however, we also found activation for mind in the eyes and rational action tasks where this was not expected.

#### TPJ for Attention Reorienting

*Theory.* According to Corbetta et al. (2008), the right TPJ is responsible for the detection of salient and behaviorally relevant stimuli in the environment that were previously unattended – a cognitive process called exogenous or stimulus-driven attention. Cabeza et al. (2012, p. 347) extended this idea by proposing the TPJ/IPL to be responsible for guiding ‘bottom-up attention by information entering working memory either from the senses or from long-term memory.’

*Predictions.* We predict attention reorienting in strategic games as players have to reorient attention away from their own goals and movements to focus on what they get to know about the other player. For false belief tasks we predict attention reorienting to take place to the same amount in the experimental condition (reorient away from own knowledge about reality to appreciate the others belief) and the control condition (reorient away from knowledge about reality to appreciate the outdated event shown the photo). Therefore, no (relative increase in) activation is predicted for false belief tasks. For social animations, rational actions, and mind in the eyes, there is no obvious competition between salient versus less salient information. Therefore we do not see any reason for bottom-up attention orienting. Trait judgment tasks often require judging whether a person habitually takes a different perspective on certain aspects of life than it is the norm. However, comparing different perspectives for a trait judgment requires attention to be devoted to two pieces of information simultaneously rather than reorienting from one to the other, so we do not expect activation in the TPJ here.

*Evaluation.* Consistent with our predictions, we found activation in right TPJ for strategic games. However, inconsistent with our predictions, we also found right TPJ activation for all five other tasks, where the attention reorienting account would not predict it.

## Conclusion

In this review we evaluated neurocognitive theories of ToM based on results from a recent imaging meta-analysis (Schurz et al., 2014). We checked whether predictions from those theories were met by the results. We made four key observations.

The first observation is that the large majority of failed predictions are due to presence of activation for ToM tasks that are not supposed to engage the cognitive processes in question. This probably shows that authors had different views on what is a ToM task and what is not. The present review is based on a “democratic” definition found in our meta-analysis (Schurz et al., 2014), i.e., we included all studies that were labeled ToM by the authors. Besides adopting such a “democratic” definition, a further strategy for future theory building could be to include tasks based on a refined analysis of specific component processes of mentalizing or teleology, rather than fixating on the umbrella term ToM (see also Schaafsma et al., 2015).

The second observation we made is that theories with best predictive accuracy (e.g., Amodio and Frith, 2006; Saxe and Powell, 2006) are often less specific about cognitive processing. For example, Amodio and Frith (2006) postulate that dorsal mPFC is engaged in all forms of mental state reflection – which is largely equivalent to being engaged in all forms of ToM. Although this claim is fully supported by our data, it does not provide a cognitive explanation of how mental state reflection (i.e., ToM) is implemented.

The third observation is that many theories use loose definitions of the regions of interest they are focusing on. This is the case both for mPFC and TPJ, and probably reflects conclusions from some literature reviews (e.g., Gallagher and Frith, 2003; Amodio and Frith, 2006; Frith and Frith, 2006; Mitchell, 2009) that the ToM network is highly consistent in localization across tasks, methods, and studies. More recently, however, reviews found that brain activation within broad areas such as TPJ or mPFC breaks apart for different ToM tasks (e.g., Gobbini et al., 2007; Bahnemann et al., 2010; Schurz et al., 2014). Thus, the predictive power of theories can be improved by reference to more specific brain anatomy. We speculate that this could also be helpful for ruling out some of the unexpected findings of activation that were not foreseen in cognitive theories (as described in our first observation).

Our fourth observation relates to the role of control conditions. The meta-analysis (Schurz et al., 2014) on which we build this review grouped ToM tasks not only by stimuli and instructions presented in the experimental condition, but also by the kind of control condition employed. Reviewing the ToM theories made clear that some accounts focus exclusively on explaining the processes taking place in the experimental condition (i.e., the ToM condition), without taking into account which processes are controlled for by the control condition. A prominent example for this is the attentional reorienting account of TPJ function in ToM (e.g., Corbetta et al., 2008). This account focuses on the false belief task to re-interpret TPJ functioning during ToM. The argument is that in the false belief task, participants first form a representation of another person’s belief and then get to know that the object of that elief has changed in reality. Therefore, when participants are later asked about the belief of the person, they must reorient their attention away from their own knowledge about reality and toward the person’s false belief. An important issue for this theory now comes with the control condition used in the analyzed false belief studies. In the false photo control condition, participants are asked what is shown on a photograph of a previous situation. One can similarly argue that, in order to answer the question, participants must reorient attention away from their knowledge of the current state of affairs and toward the past state that is shown on the photograph. Therefore, when considering brain activation differences for false belief > false photo, we do not see attentional reorienting as a straightforward explanation, since attentional reorienting seems to be needed in both conditions.

Taken together, these four observations show where existing theories fail to predict results and discuss possible reasons. This evaluation points out areas of improvement for future models.

## Author Contributions

MS and JP wrote this review article.

## Conflict of Interest Statement

The authors declare that the research was conducted in the absence of any commercial or financial relationships that could be construed as a potential conflict of interest.
